# Impact of Hanging Motionless in Harness on Respiratory and Blood Pressure Reflex Modulation in Mountain Climbers

**DOI:** 10.1089/ham.2018.0089

**Published:** 2019-06-21

**Authors:** Francesca Lanfranconi, Alessandra Ferri, Luca Pollastri, Manuela Bartesaghi, Massimiliano Novarina, Giovanni De Vito, Egidio Beretta, Lucio Tremolizzo

**Affiliations:** ^1^ School of Medicine and Surgery, University of Milano-Bicocca, Monza, Italy.; ^2^ Foundation Monza and Brianza for the Mother and Her Child, Monza, Italy.; ^3^ Institute for Health and Sport, Victoria University, Melbourne, Australia.; ^4^ PENTAVIS, Laboratory of Sport Medicine, Lecco, Italy.

**Keywords:** harness hang syncope, inspiratory elastic power, pattern of breathing, respiratory and cardiovascular reflexes, suspension trauma

## Abstract

Harness hang syncope (HHS) is a risk that specifically affects safety of harness users in mountain climbing.

***Aims:*** To evaluate individual patterns of breathing resulting from deranged cardiovascular reflexes triggering a syncopal event when a mismatch between cerebral O_2_ demand and supply is present.

***Results:*** Forty healthy participants [aged 39.1 (8.2) years] were enrolled in a motionless suspension test while hanging in harness. Respiratory gas exchange values were analyzed to assess the pattern of breathing (EpInW_el_, respiratory elastic power) and cardiovascular parameters were monitored (BP, blood pressure). Four participants experienced HHS after 30.0 (7.6) minutes, with an early manifestation of loss of control of both a sustainable EpInW_el_ and BP, starting after 10–12 minutes. Among the other participants, two different reactions were observed during suspension: (1) group G1 tolerated 32.7 (11.4) minutes of suspension by a favorable adaptation of the EpInW_el_ and BP parameters and (2) group G2 showed significantly shorter time of suspension 24.0 (10.4) minutes with unfavorable increase in EpInW_el_ and BP.

***Conclusions:*** Greater resistance to HHS occurs in people developing less marked fluctuations of both respiratory and cardiovascular reflex responses. Conversely, wider fluctuations both in control of EpInW_el_ and BP were observed in the event of decreased suspension tolerance or in syncopal events.

## Introduction

Awide knowledge gap exists regarding the risks of hanging motionless in harness, even among experienced users. The most distinctive effect is commonly known as suspension trauma, or more properly referred to as harness hang syncope (HHS). If prompt adjustment of posture/condition is not performed, a lethal multivisceral hypoxia arises even in the absence of a specific trauma leading to HHS (Seddon, 2002; Van Lieshout et al., 2003). Our recent study on the pathophysiology of HHS suggests that the condition that may jeopardize cerebral hypo-oxygenation develops within a few minutes after suspension (Lanfranconi et al., 2017b).

The most evident effect reported in this study is the existence of a critical amount of cerebral deoxygenation, as ascertained by a brain near-infrared spectroscopy investigation: cerebral deoxygenation eventually inhibits the cardiovascular reflexes while abruptly triggering syncopal event. Death due to syncope while hanging in harness could be the unwanted, predictable consequence of a delayed rescue in HHS.

**FIG. 1.**
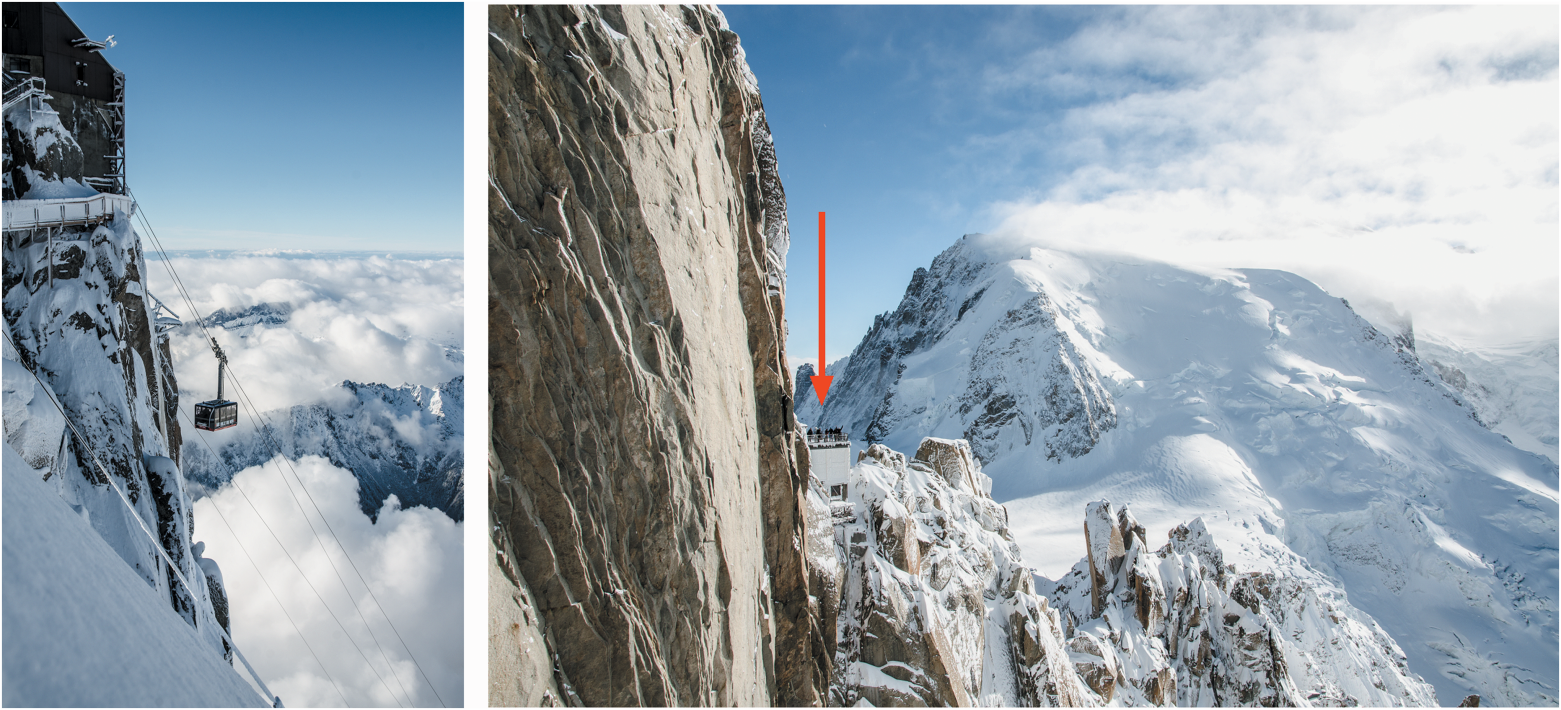
*Red arrow*: people looking at the Mont Blanc du Tacul from Aiguille du Midi, 3842 m above sea level. In the *small square*, the last section of the *téléphérique* from Chamonix (France) (photography by Ugo De Berti).

The multifaceted HHS phenomenon and its consequences are not well known by veteran climbers. Awareness about HHS is limited, and the theoretical analysis of its effects is discussed only in a few scientific articles. The consequence is an underestimated risk of life-threatening events even among experienced members of the mountaineering community (Lee and Porter, 2007; Turner et al., 2008; Mortimer, 2011; Pasquier et al., 2011; Hsiao et al., 2012). The number of high mountain casualties is increasing in Alpine regions, and HHS could potentially affect more and more harness users. Indeed, the Alpine peaks are easily accessible by *téléphériques* (aerial tramway) that can take climbers up to the first 3000 m or so, and as a result in Mont Blanc region alone 20,000-plus people attempt to reach the summit yearly (Fig. 1).

Inexperienced climbers, relying on the skills of Alpine guides to get up and down the mountain safely, are quite substantial in this impressive number of summit hikers (Wallace, 2012). This circumstance seems far more significant than it is even thought because there is only a remote chance that knowledge about high-altitude health risks, including the HHS occurrence, and countermeasure skills could be part of the cultural heritage of the thousands of climbing apprentices. At least, improving the knowledge of experienced climbers by enlightening them about measures to counteract HHS will help reduce the risk and pave the way toward a sensible and balanced approach to high mountain climbing.

Examining metabolic impairments along the human O_2_ transport utilization chain by inducing syncope, while wearing harness, could provide important insights into the multivisceral hypoxia that distinguishes HHS itself. Included in the definition of syncope are the strong relationship between gravitational stress, vasomotor failure and/or bradycardia, reduced venous return, and fallen cardiac output with a definitive decrease in cerebral oxygenation before fainting (Moya et al., 2009). Microgravity effects on cardiovascular reflexes are new findings incorporated to the understanding of the HHS pathophysiology in our previous work (Lanfranconi et al., 2017b). The next phase, in this step-by-step approach to the intriguing complexity of HHS events, concerns the involvement of the respiratory system control in triggering a syncopal event when an incompatibility between cerebral O_2_ demand and supply is present (Convertino et al., 1994, 2009; Serrador et al., 2000, 2006).

Certainly, while hanging in harness, two main conditions can interfere with the individual pattern of breathing: (1) thoracic constraint due to mechanical limitation of either the rib cage or the abdominal wall and (2) cerebral hypoxia impacting the respiratory control centers in the brain. This article is a further analysis of data originating from the same participants of our already mentioned experimental set (Lanfranconi et al., 2017b). It aimed on the capacity of the human body to cope with the effects of motionless suspension from the respiratory control's perspective. Our investigation is intended to verify if the pattern of breathing varied among participants hanging in harness. Additionally, the individual strategy to cope with cerebral hypoxia was explored to evaluate possible factors of resilience to make O_2_ available to the cerebral cortex.

## Methods

### Participants

The study conformed to the standards set by the latest revision of the Declaration of Helsinki, and all procedures were approved by the ethics committee of the University of Milano-Bicocca (approval number: 4566/2010). Personal data were treated according to standard principles of confidentiality. Participants were informed about the risks of developing HHS and the ability of the medical team to manage possible emergency. All participants signed an informed consent to be part of the project.

Forty adults participated in this study with a mean (SD) age of 39.1 (8.2) years, body mass index of 24.2 (3.03), and 85% were males and 15% females. Six additional young, healthy, and sedentary participants (gender matched, body mass indexes within the normal range) participated in an extra spirometric evaluation to estimate the impact of different mechanical loads on the respiratory system (LOAD group). See the Respiratory Evaluation section for further information. Smokers were not included in this research program.

### Suspension protocol

An extended version of the suspension test (ST) protocol has already been described in our previous article (Lanfranconi et al., 2017b). Pulmonary ventilation (${\dot V}$E, in BTPS), O_2_ uptake (O_2_) and CO_2_ output (CO_2_), and pulmonary end tidal pressure of O_2_ and CO_2_ (Peto_2_ and Petco_2_) were determined breath-by-breath using a computerized metabolic cart (K4; Cosmed, Rome, Italy). After 4–6 minutes in resting condition, each participant underwent an ST, hanging from a ventral chest ring attachment raised about 50 cm from the floor. Participants wore a full body harness with ergonomic back padding and leg-loops with quick-release buckles (Golden Top Evo Alu; CAMP, Premana, Italy). The ST time was recorded from the start in motionless hanging condition (reached within 20–40 seconds of hanging in harness) until the time the participant returned to the ground.

Participants who developed syncope were kept in a supine position by passively raising both their legs above cardiac level (Bridges and Jarquin-Valdivia, 2005; Kweon et al, 2012). No time limit was previously set. In the case of syncope, the end time of the ST was when the unconscious participant laid down on the examination table.

### Respiratory evaluation

#### Pulmonary function tests

Each participant was asked to perform a spirometry test, sitting and in resting condition (Vmax Spectra 229; SensorMedics, Yorba Linda, CA). The variables measured were as follows: the volume from a maximal forced expiratory effort (FVC, the forced vital capacity), the forced expiratory volume in the first second (FEV1), the forced expiratory flux at 25%–75% (FEF25–75), and the peak expiratory flow rate. A visual evaluation of the expected spirometry curves was performed, and the average of three consecutive and repeatable measures was used. All variables were expressed with respect to predicted values according to age, sex, and anthropometric values.

On another day, the LOAD group performed extra spirometry measures to estimate the impact of different mechanical loads on the respiratory system: (1) in resting and sitting orthostatism; (2) in clinostatism, lying on the examination table; (3) while hanging in the harness; and (4) while sitting and applying a pneumatic tourniquet cuff (15 cm height, 120 cm length) around the upper abdomen/lower chest, inflated at progressively increased pressure (25–50–75 mmHg).

#### Respiratory power

A simple approach to estimate the inspiratory elastic power was recently proposed and proved useful to compare the energy expenditure when changing the pattern of breathing due to respiratory diseases or environmental conditions (Dellweg et al., 2008; Passoni et al., 2015; Beretta et al., 2017). Considering the linearity of the overall volume–pressure relationship of the respiratory system, between the resting respiratory capacities up to the end-inspiration, the elastic/resistive work performed by the inspiratory muscles (InW_el_) can be defined as the area of the right triangle where one leg (cathetus) is the change in lung volume or tidal volume (V_t_), and the other leg is the corresponding applied pulmonary pressure (ΔP), namely:

(1) InW_el_ = ½(V_t_*ΔP).

Being the hypotenuse, the total respiratory compliance or C_res_ = V_t_*ΔP^−1^ one has:

(2) InW_el_ = ½(C_res_)^−1^*V_t_^2^

and knowing the respiratory rate (R_f_), the InW_el_ per minute becomes the inspiratory elastic power (Ep):

(3) EpInW_el_ = ½(C_res_)^−1^*V_t_^2^*R_f_.

Arbitrarily assuming the factor C_res_ unchanged during suspension, the product ½V_t_^2^*R_f_ can provide an index of the relative changes in EpInW_el_, when comparing different patterns of breathing.

### Study design and statistical analyses

This was a single-center, analytic observational study, with cross-sectional design. Values are expressed as mean (SD). Sample size calculation determined that a sample of 34 subjects would be adequate to detect a difference of 8 minutes in suspension time between the participants, with a power of 0.80 (α = 0.05). D'Agostino and Pearson omnibus normality test was used to check if the values come from a Gaussian distribution: all the variables passed the normality test. The statistical significance of the difference between mean values, considering the different circumstances, was evaluated by: (1) ordinary one-way ANOVA, followed by Tukey's multiple comparison test, with a single pooled variance and (2) unpaired *t* test, two-tailed. Regression analyses were performed using the least squared residuals method. The level of significance was set at *p* < 0.05.

All statistical analyses were performed using a commercially available software package (Prism 6.0; GraphPad, La Jolla, CA).

## Results

### Pattern of breathing during the ST

Four participants experienced syncopal event after 30.0 (7.6) minutes (SYN). Within SYN, one person was a regular user of harness.

**FIG. 2.**
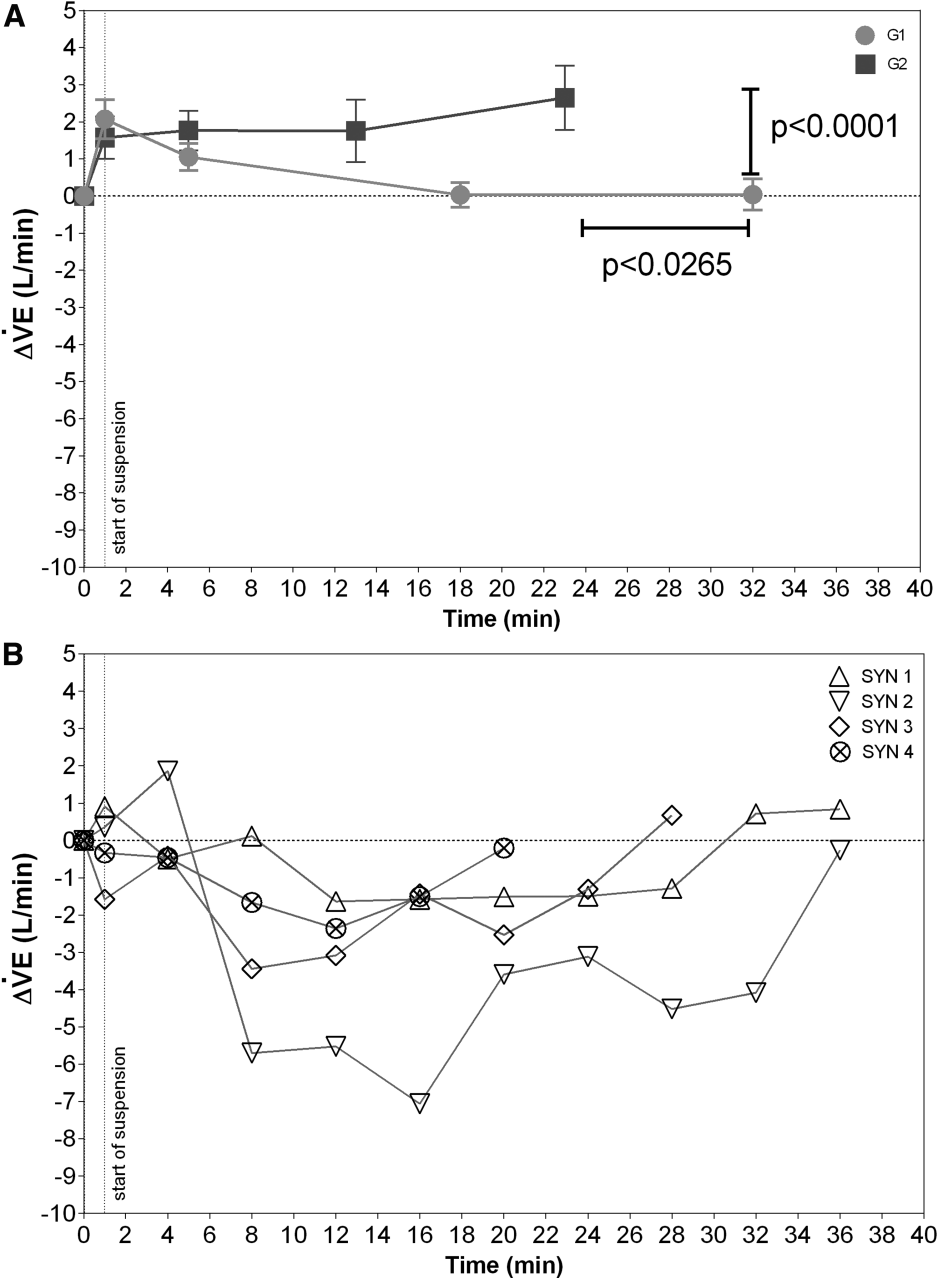
Time courses of changes in ventilation (${\dot V}$E) values during the suspension test in groups 1 and 2 (G1 and G2, respectively) **(A)** and in the four participants who experienced the harness hang syncope (SYN) **(B)**.

*A posteriori*, the other participants were divided into two groups, based on the time course of ${\dot V}$E and its subcomponents during the ST, identified as G1 and G2 (*n* = 22 and 14, respectively). As shown in Figure 2A, in G1, a transitory increase in ${\dot V}$E was observed and gradually decreased after about 15 minutes. In G1, different reasons led to the end of the ST including diastolic hypertension, subjective poor harness comfort, trembling, sweating, severe systolic hypertension, heart rate increase from baseline value of >25 bpm, and headache. In G2, although ${\dot V}$E similarly increased as in G1 when suspension started, it remained high during all the STs that were terminated when systolic blood pressure (SBP) reached values over 160 mmHg and/or one or more of the following signs were indicated: trembling, sweating, nausea, headache, and light-headedness. General respiratory “fatigue” was reported by many of the G2 group participants.

Suspension time was significantly shorter in G2 [24.0 (10.4) minutes] compared with G1 [32.7 (11.4) minutes]. At the end of the ST, ${\dot V}$E was significantly higher in G2 [14.3(0.81) L/min] compared with G1 [10.1 (0.41) L/min].

An initial variable response of ${\dot V}$E was observed in SYN (Fig. 2B) early on right after suspension, followed by a decrease that tended to return to normal approaching the final syncopal event, which occurred at about 30.0 (7.6) minutes. Clinical characteristics of HHS were as follows: (1) abrupt onset (few seconds) in two participants, with only evanescent symptoms before the loss of consciousness and (2) slower onset (from seconds to minutes) in two participants, with nausea and light-headedness before syncope. Severe harness discomfort before HHS was experienced by three participants not used to wearing harness.

**FIG. 3.**
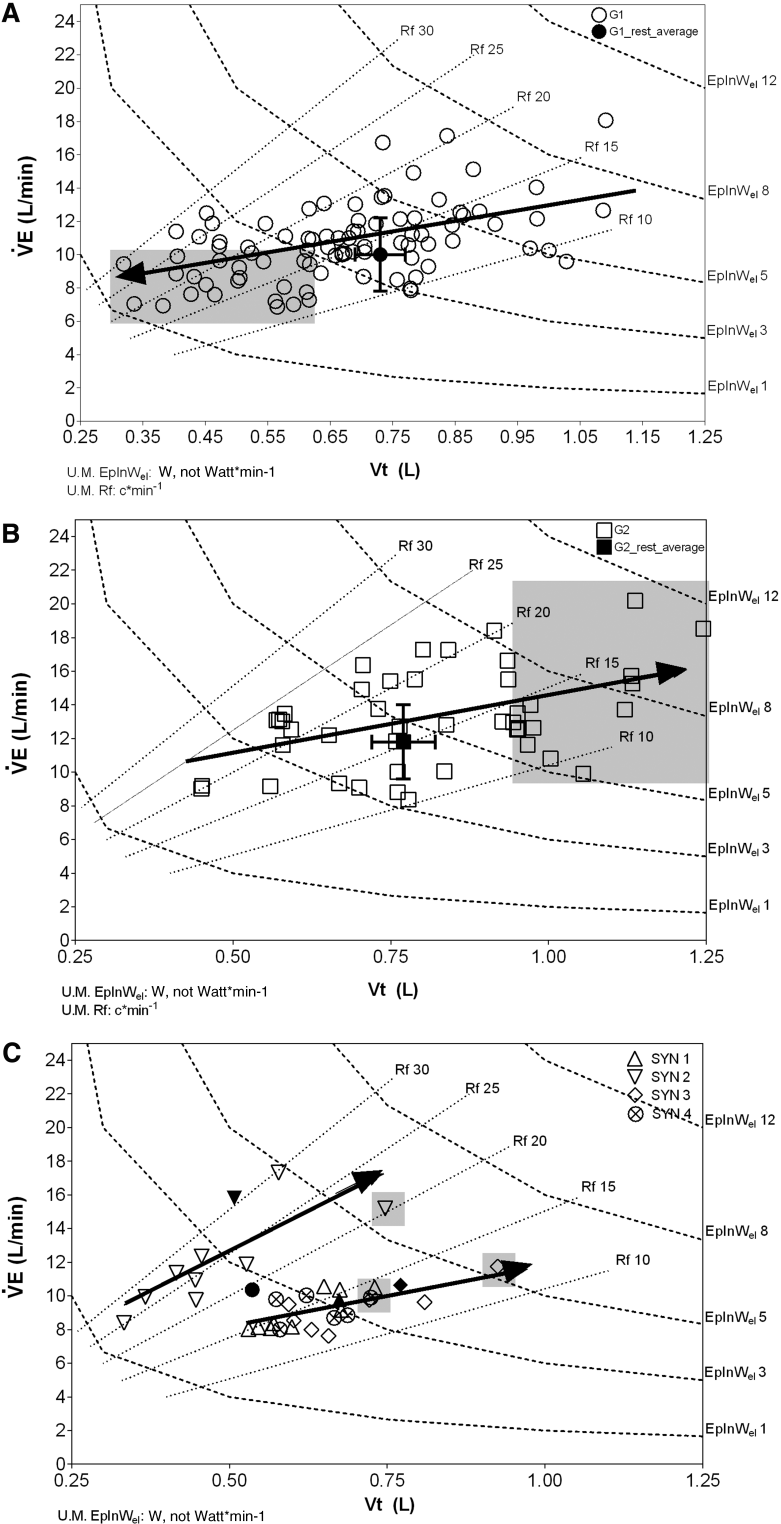
Pattern of breathing during the suspension test in G1 **(A)**, G2 **(B)**, and SYN **(C)**. Each participant is showed at resting, 25%, 50%, and 100% of the suspension test time. *Full symbols* indicate the group average values at baseline. The *gray* area is where the individual final points fall. The averaged regressions between tidal volume (V_t_) and ventilation (${\dot V}$E) values are presented, with the *arrows* indicating the direction of variation from baseline. Isopleths of respiratory frequency (R_f_) and inspiratory elastic power (EpInW_el_) are indicated.

Figure 3 presents the changes in the pattern of breathing among the groups, during the ST, as described by the plot of ${\dot V}$E versus V_t_. The graphs also show isopleths of respiratory frequencies (R_f_, from 10 to 30 c/min), corresponding to the range from rest to high-intensity exercise in humans. The isopleths of inspiratory elastic power (EpInW_el_, right ordinate, from 1 to 12 W) were also displayed.

In G1 (Fig. 3A), due to the initial increase in ${\dot V}$E, values were first distributed to the right of the average resting value (closed symbol), yet, after this initial phase, as suggested by the direction of the arrow, values started appearing on the left of the graph as the end of the ST was approaching, due to the progressive decrease in ${\dot V}$E and V_t_ and an increase in R_f_. This resulted in a remarkable decrease in EpInW_el_ from ∼8 down to $\sim$1 W. This plot essentially suggests that the pattern of breathing, to achieve ${\dot V}$E, was remodeled through a decrease in V_t_ and an increase in R_f_, as shown by the arrow crossing the R_f_ isopleths from 10 toward 30 c/min.

A different pattern of breathing in response to suspension was observed in G2, as suggested by the arrow's direction (Fig. 3B): indeed, ${\dot V}$E kept increasing relative to the average resting value and this occurred with an increase in V_t_, a decrease in R_f_, and a remarkable increase in EpInW_el_ from $\sim$2 to $\sim$12 W.

Figure 3C shows the pattern of breathing of the SYN: three participants displayed the same pattern of breathing and a common arrow is shown, whereas one participant was considered individually (another arrow was displayed). In all the SYNs, there was an initial decrease in ${\dot V}$E under the resting values (full symbols), with a subsequent return toward higher values as the end of the ST was approaching. In all SYNs, this occurred with a moderate decrease in R_f_, but a remarkable increase in V_t_ was observed resulting in a considerable increase in EpInW_el_.

### Oxidative metabolism

**FIG. 4.**
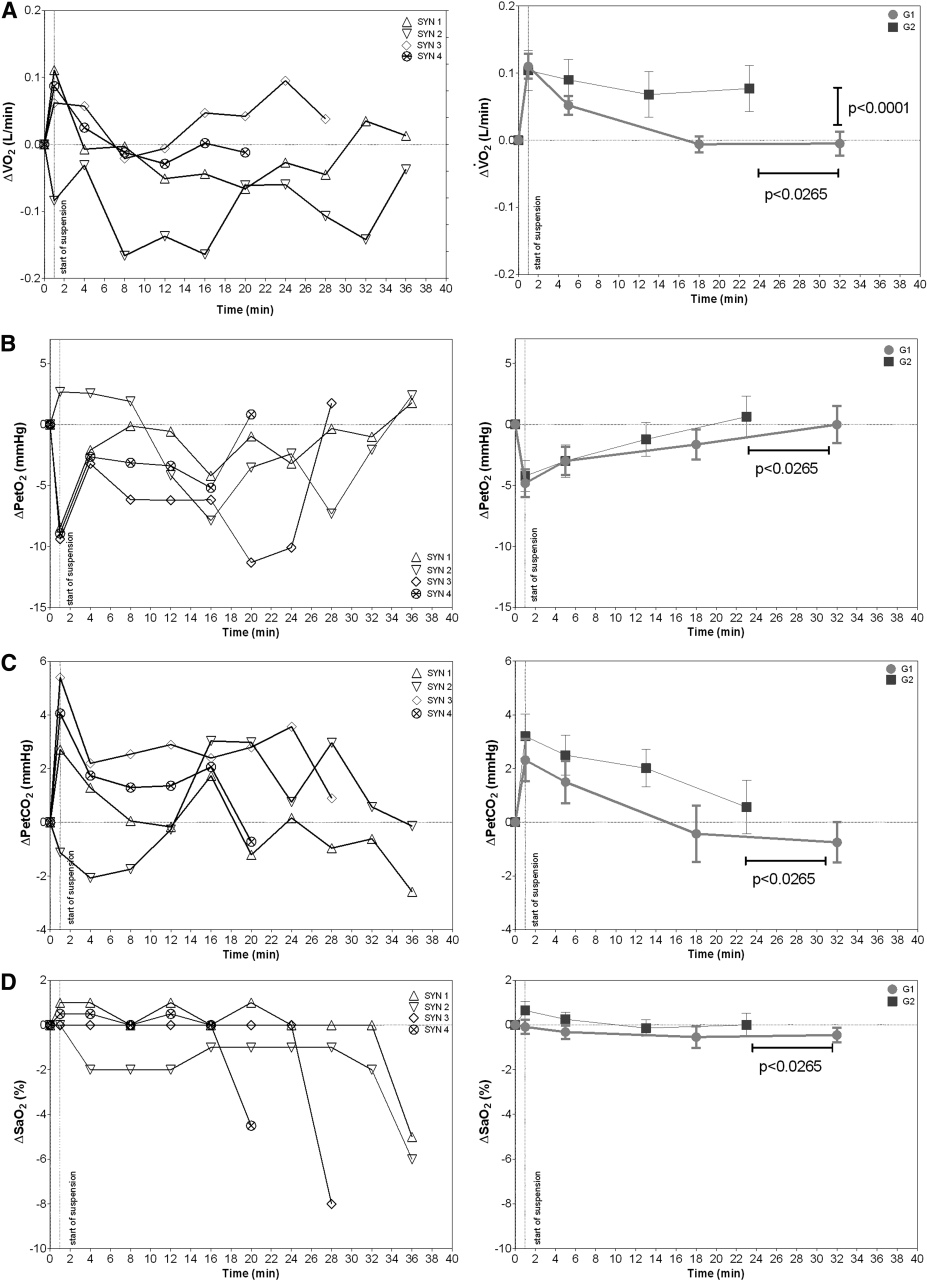
Time courses of changes in gas exchange values during the suspension test in SYN (*left panels*) and G1 and G2 (*right panels*). In **(A)**, O_2_ uptake (${\dot V}$O_2_) time courses. In (**B** and **C**), pulmonary end tidal pressure of O_2_ and CO_2_ (Peto_2_ and Petco_2_) time courses. In **(D)**, arterial blood O_2_ saturation (SaO_2_) time courses.

Figure 4A (right) shows a sudden increase in ${\dot V}$O_2_ at the onset of suspension in G1, waning within about 15 minutes and remained still, until the end of the ST. Conversely in G2, ${\dot V}$O_2_ increased at the onset of suspension and then tended to remain high up to the end of suspension. At the end of the ST, ${\dot V}$O_2_ was significantly lower in G1 [0.30 (0.07) L/min] compared with G2 [0.43 (0.08) L/min]. Peto_2_ decreased in both G1 and G2 at the onset of ST, in line with the increase in ${\dot V}$E, returning thereafter toward normal (Fig. 4B, right). The changes in Petco_2_ were in the opposite direction within respect to Peto_2_ for both G1 and G2 and waned overtime (Fig. 4C, right). The changes in SaO_2_ were well within 1% in both G1 and G2 for the entire duration of the ST (Fig. 4D, right).

In SYN, a variable response at the onset of suspension was documented on average; a decrease in ${\dot V}$O_2_ was observed with a return toward normal values approaching the syncope (Fig. 4A, left). Peto_2_, decreased between 15 and 25 minutes and increased toward normal only in the late phase preceding syncope, paralleling the increase in ${\dot V}$E (Fig. 4B, left). The changes in Petco_2_ were in opposite direction compared with Peto_2_ and waned overtime (Fig. 4C, left). In SYN, SaO_2_ remained unchanged up to the harsh decrease preceding syncope (Fig. 4D, left). SaO_2_ during syncope was statistically lower compared with G1 and G2.

### Pulmonary volumes and lung diffusion capacity during suspension

Table 1 shows spirometry measures in resting condition (before wearing the harness), which did not differ among groups (SYN, G1, G2, and LOAD). Table 1 also shows the same variables, for the LOAD group, when the impact of chest loads was due to change in posture (clinostatism), suspension ($\sim$15–20 minutes), and mechanical restriction of the thoracic–abdominal region of participants. In this group, only the clinostatism and chest–abdominal wall strapping (50–75 mmHg) resulted in a 6%–12% significant decrease of air flow-dependent parameters. The 75 mmHg chest–abdomen wall strapping was described by all participants as annoying, to some extent definitely more restrictive than wearing harness in suspension.

**Table 1.** Pulmonary Function Test Parameters for Groups G1, G2, SYN, and LOAD

		*FVC*	*FEV1*	*Tiffeneau*	*FEF25–75*	*PEF*
		*% of predicted*	*% of predicted*	*%*	*% of predicted*	*% of predicted*
G1, RESTING						
Orthostatism	Mean	119.60	121.90	84.40	117.93	113.35
	SD	11.50	9.90	5.20	21.87	24.04
G2, RESTING						
Orthostatism	Mean	121.39	123.88	84.14	113.12	129.96
	SD	7.57	11.89	5.01	23.22	15.39
SYN, RESTING						
Orthostatism	Mean	129.94	131.19	83.82	114.84	136.81
	SD	19.31	17.49	5.39	13.52	10.59
LOAD group						
Orthostatism	Mean	114.36	113.15	82.73	107.87	115.13
	SD	9.96	12.81	3.76	28.13	18.95
Clinostatism	Mean	108.35^*^	104.63^*^	80.79	94.40^*^	104.50^*^
	SD	11.46	12.36	2.63	19.21	20.51
Suspension	Mean	111.31	111.86	84.17	104.85	117.02
	SD	13.41	14.35	4.39	27.21	17.91
Chest/abdominal strap	Mean	109.00	110.24	84.72	107.84	108.99
25 mmHg	SD	14.14	14.76	4.24	24.09	22.77
Chest/abdominal strap	Mean	107.58^*^	106.73^*^	82.90	102.13	104.86^*^
50 mmHg	SD	11.79	14.48	2.82	24.05	28.00
Chest/abdominal strap	Mean	108.02^*^	107.44^*^	83.21	101.23	103.45^*^
75 mmHg	SD	8.03	10.05	2.82	18.16	22.29

Orthostatism: sitting at rest; clinostatism: lying on the examination table; suspension: while hanging in the harness; chest/abdominal strap: while sitting and applying a pneumatic tourniquet cuff inflated at progressively increased pressure (25–50–75 mmHg).

^*^ *p* < 0.05 with respect to orthostatism in the LOAD group.

FVC, forced vital capacity; FEV1, forced expiratory volume in 1 second; FEF25–75, forced expiratory flux at 25%–75%; PEF, peak expiratory flow rate.

### Cardiovascular and respiratory control

**FIG. 5.**
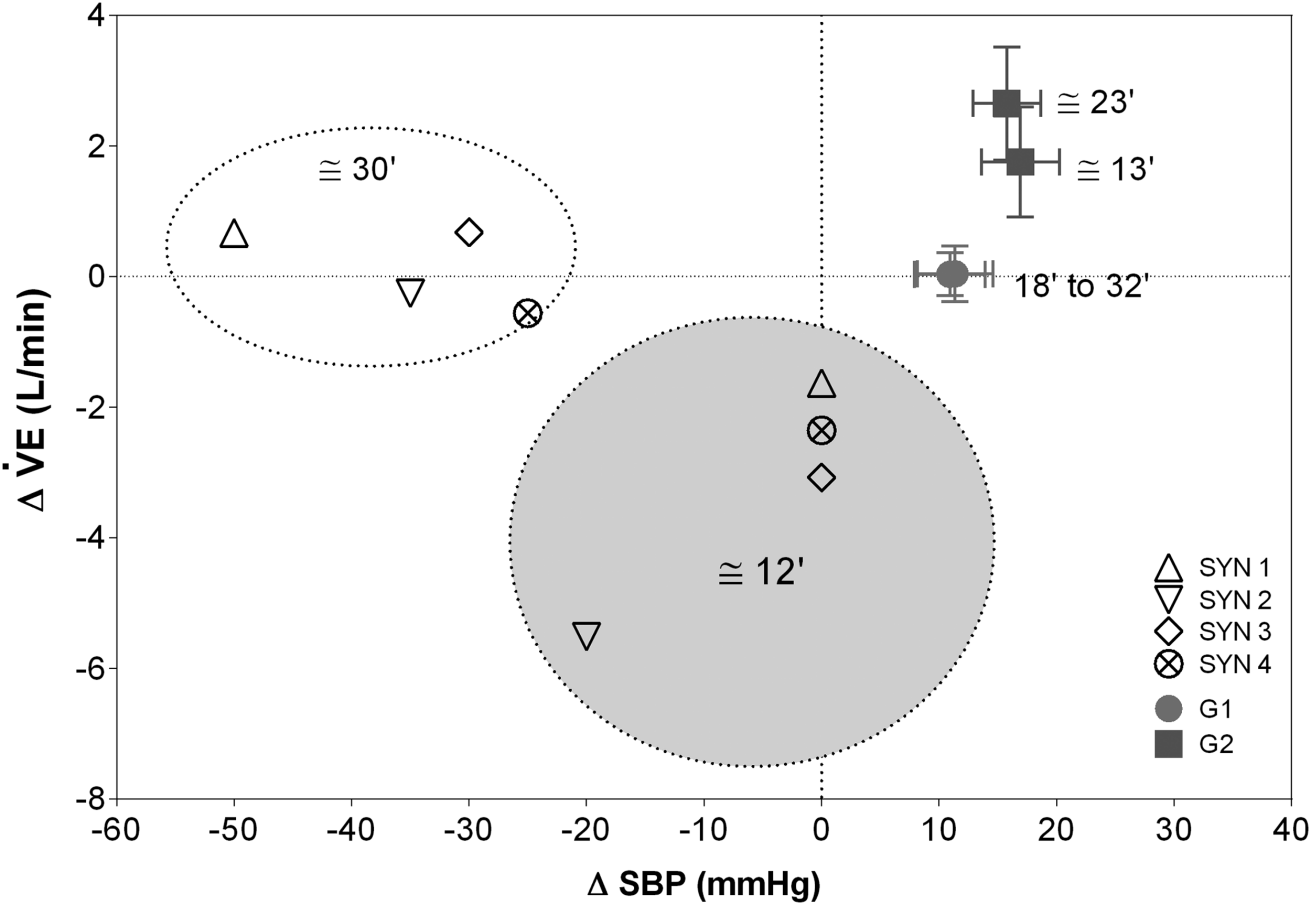
Relationships between variation of ventilation (Δ${\dot V}$E) and systolic blood pressure (ΔSBP) from baseline values in G1, G2, and SYN. Values are presented at two specific time points from the start of suspension: 50% (12, 13, and 18 minutes for SYN, G2, and G1, respectively) and the end of suspension (30, 23, and 32 minutes for SYN, G2, and G1, respectively). The SYN values are presented individually.

Figure 5 shows the average fluctuations in SBP plotted versus ${\dot V}$E changes in G1, G2, and SYN. The plot suggests different patterns of cardiovascular and respiratory controls, at two average time points during the ST (reported in minutes), corresponding to the middle and the end of suspension. In G1, ${\dot V}$E remained essentially unchanged at the two time points while SPB increased slightly by $\sim$10 mmHg. In G2, progressive increases in ${\dot V}$E and SBP were observed during the ST. In the four SYN, decrease in ${\dot V}$E and/or SBP occurred at an earlier time point after the ST (about 12′, 40% of the ST time), while at the end of the ST (30′), the dominant factor was a dramatic decrease in SBP and the ${\dot V}$E tended to return toward basal values.

## Discussion

The pathophysiology of HHS depends on factors interfering with cerebral oxygenation, including the gravity-dependent pooling of blood and interstitial fluid in the lower parts of the body along with the deterioration of cardiovascular reflexes. This experiment helps us expand our knowledge about the potential effects of cerebral hypoxia by evaluating the impact of HHS on the pattern of breathing and respiratory reflexes. The study was performed on a relatively large number of healthy individuals, compared with previous studies on HHS, which gave us the opportunity to evaluate the individual variability in the pattern of breathing during the ST.

The results of the investigation suggested that participants who did not develop HHS were able to activate remedial responses through the O_2_ transport and utilization chain of various intensities to defend brain oxidative metabolism needs. Conversely in SYN, suspension triggered an imbalanced response of respiratory and cardiovascular reflexes leading to critical cerebral hypoxia.

### The pattern of breathing

Severe hypoxia can be individually sustained by gas exchange and pH compensation, until a critical point of cerebral hypoxia is reached causing syncope (Subudhi et al., 2007; Convertino, 2014; Lanfranconi et al., 2017b). A crucial point in control of ${\dot V}$E is the ability of the respiratory centers to set a pattern that is the most adequate to fulfill the actual O_2_ demand by targeted tissues, where failing to control ${\dot V}$E leads to a reduced compliance in facing different situations as observed in respiratory inefficiency in patients with motor neuron disease (Lanfranconi et al., 2017a). It appears that the ST triggered a respiratory response that varied among participants, suggesting different strategies to activate sensory inputs as well as higher hierarchical controls of ${\dot V}$E.

In G1, the strategy allowing alveolar gases to be kept unaltered was characterized by a progressive shift of ${\dot V}$E toward a less demanding pattern of breathing, that is, decreasing V_t_ and increasing breathing frequencies. In G2, alveolar gas pressures as well as O_2_ blood saturation were also maintained, but the respiratory response appeared to be less efficient as the pattern of breathing implied a greater energy cost due to the increase in V_t_ (Fig. 3). In SYN, the pattern of breathing was the most unpredictable and ungainly as it led to: (1) wide oscillations in alveolar gas pressures, (2) a higher respiratory energy cost, and ultimately, (3) progress toward a remarkable desaturation of blood preceding fainting.

### Metabolic cost of the pattern of breathing

Indeed, the sharp increase in ${\dot V}$O_2_ observed in G1 and G2 and in three of the four SYNs at the start of the ST is an interesting phenomenon that reflects a shared response leading to the same changes in alveolar gas composition (Fig. 4). As it occurred shortly after the start of the ST, the augmented O_2_ in this phase should be considered an effect of an acute hyperventilation response due to a very low increase of oxidative metabolism rate. Congruently, the changed posture from resting condition to the suspension posture (hanging in the harness) generated the recruitment of the air gases stored in the death space of conducting airways with a consequent increased Petco_2_ and decreased Peto_2_, as soon as the ${\dot V}$E augmented.

This event, on a larger scale, is otherwise observed in the very first phase of an incremental exercise test when the rising of V_t_ is the main way to increase ${\dot V}$E. Another additional explanation for the observed changes in expired gas composition (Peto_2_ and Petco_2_) at the onset of the ST is that the hyperventilation response acted by preferentially emptying the lungs apex, relatively less perfused, because of restricted chest movement imposed by hanging in harness and subsequent constraint on diaphragmatic regions (Pulletz et al., 2010).

Otherwise, on the subsequent phases of the ST (after $\sim$4–6 minutes from the start), different patterns of adaptation were evident between groups indicating a range of responses varying from coping well with the suspension in harness to syncope. The transitory change in ${\dot V}$O_2_ disappeared in G1, while in G2, a true increase in metabolic needs by recruiting respiratory muscles was evident by the maintained level of ${\dot V}$O_2_ during the ST. In G2, the increased ${\dot V}$E and the shorter time of suspension compared with G1 participants might be envisaged as warning signals of subcritical level of cerebral hypoxia producing an inhibition of rhythmogenesis at the brainstem. It is to be noted that a remarkable respiratory distress was experienced by G2 participants at the end of the ST, with an increased respiratory energy expenditure reflecting the choice of an uneconomical pattern of breathing.

Essentially, no such excitatory respiratory reflex was observed in SYN where a full inhibition of respiratory drives was found.

### Mechanical constrains during suspension in harness

There is a general misinterpretation that wearing a harness, also when it perfectly fit the size of the climber/worker, limits the physiological lung ventilation by generating an external thoracic restriction. On the contrary, we are not prone to consider the harness by itself as cause of a potentially degenerated pattern of breathing. It seems that the ST caused a reflex respiratory response implying the intervention of respiratory centers, at brainstem level, due to specific sensory inputs. More to the point, it was not the harness model by itself the cause of an inconsistent pattern of breathing, but the sudden postural orthostatism with changes in the force distribution applied to the chest wall that payed a tribute in term of thoracic distortion. In case one is sitting on a chair with the harness, he/she does not experience any mechanical limitation of the chest wall. In fact, since spirometry forced dynamic air flow measurements were essentially unaffected by the ST (Table 1), one may otherwise speculate about a potential role of prolonged chest wall distortions as sensory triggers (from chest wall mechanoreceptors) affecting the reflex pattern of breathing during the motionless suspension (Van Noord et al., 1986; Nishino et al., 1992).

Certainly, the response to this sustained postural chest wall distortion in G1, that is, ${\dot V}$E maintained through an economic pattern of breathing, has been previously described as a spontaneous physiological reflex responding to mechanoreceptors activation in conditions where a certain degree of rib cage and abdominal restrictions were applied (Van Noord et al., 1986; Nishino et al., 1992). In our experimental set, it takes ∼4–6 minutes of the ST before the pattern of breathing was activated by this mechanoreceptors stimulation. In G2, the constant mechanoreceptors firing appeared to raise a different strategy to increase the neural respiratory drive needed to achieve any given ${\dot V}$E during the ST, probably due to a more complex regulation by the superior hierarchical controls of breathing when triggered (Cassandra et al., 2014).

The respiratory centers located in the brainstem, especially at the level of catecholaminergic neurons projecting to the paraventricular nucleus of the hypothalamus, might contribute to modulate the cardiorespiratory and neuroendocrine responses to hypoxia (Dampney et al., 2003; King et al., 2015).

An interesting consideration is related to the reduced spirometry values when the 50 and 75 mmHg chest–abdominal wall strappings were applied (Table1): from ergonometric perspectives, given the relatively short time for developing HHS, we might recommend that harnesses should be designed to assure full rib cage and diaphragmatic expansion to minimize upper abdominal and lower chest wall constriction. Indeed, in case one wears a harness that do not fit his/her size being too tight, the effect of suspension adds up to the consequence of postural harness restriction (i.e., augmented V_t_ and reduced FVC and FEV1, respectively). A severe suspension-related dyspnea should be induced also if the severity of restricted spirometry at rest may be mild (Mendonca et al., 2014).

### Suspension marks respiratory and blood pressure controls

The heterogeneity of the pattern of breathing in response to significant progressive cerebral hypoxia during the ST supports the fact that monitoring both cardiovascular and respiratory responses might provide useful predictors of individual trends toward less stable conditions of hemodynamic resiliency (Fig. 5). One may hypothesize that the difference in reflex cardiorespiratory response mostly reflects a change in sensory input as well as in brainstem processing and integration. The corresponding cardiac and respiratory control centers are anatomically in close proximity and physiologically widely interconnected. Convertino et al. (2009) suggested that although respiration is usually associated with gas exchange, acid–base balance, endocrine, and immune functions, a less appreciated function is the increase in venous return and cardiac filling due to a “respiratory vacuum pump” effect, resulting from the negative intrathoracic pressure created during inspiration.

This compensatory heart–lung interaction appears to be a natural mechanism in response to circulating volume reduction as seen in heart attacks: exacerbation of the respiratory pump by spontaneous gasping (elevated pattern of breathing) has been reported to occur in response to cardiac pump arrest and hypovolemia, where it has been associated with successful clinical outcome (Bobrow et al., 2008). Indeed, metaboreflexes originate from gasping, when a diminished cardiac output is present, leading to a vasoconstriction activation (via sympathetic stimulation). This response generates an increased blood pressure able to maintain sufficient perfusion to the active tissues (i.e., respiratory muscles) (Miller et al., 2002). Thus, in G2 and SYN, the hyperventilation response seems to be a reaction to cope with critical levels of cerebral hypoxia and may represent an important compensatory mechanism to optimize cardiac filling and prevent BP decrease.

The case of G1 participants is consistent with the notion that peripheral vasoconstriction plays a central role in maintaining BP: Convertino (2014) found that the control of systemic vascular resistance was greater in high tolerant orthostasis participants compared with low, in line with greater elevations in the circulating vasopressor hormones (vasopressin, angiotensin, and norepinephrine). An overdriven control of both vascular and respiratory centers can lead conversely to an intolerable stress of the systems and a rapid increase of energy expenditure of respiratory muscles to maintain ${\dot V}$E, as observed in G2. In the latter, the cause of ending the ST was due to severe systolic BP value (SBP >160 mmHg or diastolic blood pressure >110 mmHg) in around 60% of cases.

Contrarily to Convertino et al. (2009), in HHS, the failure in control of both ${\dot V}$E and BP appears to be an early phenomenon already evident after 10–12 minutes of the ST when deeper breathing was associated with progressive hemodynamic decompensation. A clinical hot topic could be to set the timelines of individual trends leading to the critical point of no return, instead of pointing the attention just when “critical values” are reached.

## Conclusion

The present findings suggest that the ability to cope with hanging motionless in harness occurs in people developing less marked as well as more rapidly fading of fluctuations in both respiratory and cardiovascular reflex responses. Conversely, wider fluctuations in control of ${\dot V}$E and BP result in a progressive decrease in tolerance to suspension. In SYN, the analysis of the pattern of breathing in addition to the cardiovascular response suggests that the imbalance leading to cerebral hypoxia was an early phenomenon (10–12 minutes from the start of the ST) irreversibly ending in syncopal event.

Finally, based on our results, we recommend the more fairly term “harness hang syndrome” rather than “suspension trauma” for defining this potentially fatal condition.

### Authors' Contribution Statement

Substantial contributions to the conception or design of the work: F.L., M.B., L.P., G.D., M.N. Substantial contributions to the acquisition and analysis of data: F.L., M.B., L.P., G.C., M.N. Substantial contributions to the interpretation of data: F.L., A.F., E.B., G.D., L.T. Drafting the work or revising it critically for important intellectual content: All authors. Final approval of the version to be published: All authors. Agreement to be accountable for all aspects of the work in ensuring that questions related to the accuracy or integrity of any part of the work are appropriately investigated and resolved: all Authors.
